# Carvacrol and Thymol Content Affects the Antioxidant and Antibacterial Activity of *Origanum compactum* and *Thymus zygis* Essential Oils

**DOI:** 10.3390/antibiotics13020139

**Published:** 2024-01-31

**Authors:** Mounia Chroho, Youssef Rouphael, Spyridon A. Petropoulos, Latifa Bouissane

**Affiliations:** 1 Molecular Chemistry, Materials and Catalysis Laboratory, Faculty of Sciences and Technologies, Sultan Moulay Slimane University, BP 523, Beni-Mellal 23000, Morocco; mounia.chroho@usms.ma; 2 Department of Agricultural Sciences, University of Naples Federico II, Via Università 100, 80055 Portici, Italy; youssef.rouphael@unina.it; 3 Department of Agriculture, Crop Production and Rural Environment, University of Thessaly, Fytokou Street, 38446 Volos, Greece

**Keywords:** chemical composition, hydrodistillation, Lamiaceae, antioxidant capacity, DPPH, FRAP, antibacterial capacity

## Abstract

Essential oils are of great interest due to their potent pharmaceutical and biological activities. In this study, essential oils extracted from *Origanum compactum* and *Thymus zygis* originating from the Middle Atlas of Morocco were investigated. Their chemical compositions were analyzed using gas chromatography and mass spectrometry, while the assessment of the trapping power of the radical (DPPH: 1,1-diphenyl-2-picrylhydrazyl) and the reducing antioxidant potential of ferric ions (FRAP: Ferric Reducing Antioxidant Power) were performed in order to evaluate the antioxidant activity. Their antibacterial potency was tested against six bacterial strains through the disk diffusion method. The chromatography analyses of the extracted essential oils highlighted the presence of two main components, namely carvacrol at 75.70% in *O. compactum* and thymol at 40.67% in *T. zygis*. The antioxidant activity tests showed that both essential oils demonstrated a significant antioxidant activity comparable to the positive control (e.g., ascorbic acid). The antibacterial activity results showed a strong antimicrobial effect for both essential oils, compared to synthetic antibiotics. This study affirms the presence of bioactive components with interesting antioxidant and antibacterial activities in the essential oils extracted from *Origanum compactum* and *Thymus zygis*, which could find several applications in the food and pharmaceutical industries through the substitution of synthetic antioxidants and antibiotics.

## 1. Introduction

Essential oils (EOs) are secondary metabolites of plants that contribute to their distinct aroma. They are made up primarily of volatile terpenes and hydrocarbons. Essential oils are generated in over 17,500 aromatic plants and are located in different parts of the plants such as flowers, fruits, seeds, and leaves, in addition to the woods, roots, and rhizomes [1]. Currently, there are more than 3000 essential oils identified in plants with 300 of them having significant economic value, particularly for the sanitary, pharmaceutical, agronomic, cosmetic, food, and fragrance industries [2]. Essential oils are an important source of bioactive compounds. Some of them are associated with specific therapeutic qualities and they are believed to be able to prevent or even treat some organ diseases or systemic illnesses [3,4]. Numerous studies have been published corroborating significant activities of essential oils including antimicrobial, antibacterial, antifungal, antiviral, antioxidant, anxiolytic, antidepressant, anticancer, anti-inflammatory, anesthetic, and reducing the blood cholesterol level [1,5]. Among the interesting properties of essential oils, the antioxidant and antibacterial activities are highly appreciated since they might help to address two significant issues affecting human health, namely oxidative stress and antibiotic resistance [6]. In fact, they can act as natural antioxidants and antibiotics and substitute the synthetic ones that might be noxious and unhealthy [7]. The essential oils of plants are a complex combination of chemical components. Each component has unique physical and chemical characteristics that, when mixed in different quantities, produce a synergic effect that may give particular qualities and properties to the essential oil. Many factors can affect the composition of the essential oils. Some are intrinsic (seasonal, sexual, genetic variations, ontogenetic) and the others are extrinsic (environmental and ecological properties) [8]. The components of EOs are usually responsible for its biological properties. The latter are due to the presence of terpenoids, a combination of various 5-carbon-base (C5) with diverse carbon skeletons and various oxygenated derivatives, including phenols, alcohols, ethers, esters, and peroxides [9].

Lamiaceae is one of the main families of aromatic and medicinal plants that produce significant amounts of essential oils. It is a large family with numerous genera and species that includes oregano and thyme in addition to lavender, marjoram, sage, and peppermint. They often accumulate volatile chemicals in glandular trichomes [8]. Among the species of this family, the genus *Origanum* and *Thymus* are widely known and highly appreciated in traditional medicine throughout the world. In Morocco, both the species are included in the most significant medicinal plants commonly used in traditional medicine and mostly studied for their several biological activities [7,10,11]. Known locally as “Zaâtre” for *O. compactum* and “Zaîtra” for *T. zygis*, they may be applied against a wide spectrum of pathologies and in industrial fields such as foods, pharmaceuticals, aromatherapy and perfumes [10,11,12,13,14,15,16,17,18,19]. However, the deep overexploitation of the two species can lead to their extinction [12,13,15,16,20].

The present contribution aimed to make a comparative study to highlight the benefits of the essential oils extracted locally from *O. compactum* and *T. zygis* exploited explicitly in the Middle Atlas of Morocco. Their chemical compositions were investigated to identify the main components, and then evaluate their antioxidant and antimicrobial activities. Moreover, a correlation between components and biological activities was also established in order to pinpoint those compounds that shape the quality of the essential oils.

## 2. Results and Discussion

### 2.1. Yield of Essential Oils

Regarding *O. compactum*, the plant material had a moisture content of 9.83% yielding 3.88% of essential oil. This yield is within the same range of yield values for the same species reported in other studies [12,13,14,15,16,17]. In fact, in more than a hundred samples collected from different sites throughout Morocco, the yields obtained varied between 0.31% and 4.41% [13,21,22,23], whereas in other studies upper yields of 5.4% and 5.68% were reported [24,25].

As for *T. zygis*, the extraction yielded 1.94% of essential oil from plant tissues with a moisture content of 14.5%. This value is very close to that one obtained from the same species collected at Ait Nouh—Khenifra [26] and slightly higher than the one reported from the same species collected at Ait Yahya—Khenifra (1.55%) [26]. The obtained yield was also higher than the one (1.20%) attained from samples of *T. zygis* collected at the beginning of flowering in the region of Taza [27].

Therefore, it could be suggested that several factors could explain this variation in essential oil’s yield in aromatic plants in general or between plants of the same species. Among others, the vegetative stage, geographical distribution, storage conditions, and harvest periods could be mentioned, in addition to the extraction method [28].

### 2.2. Chemical Composition of Essential Oils

**Figure 1:**
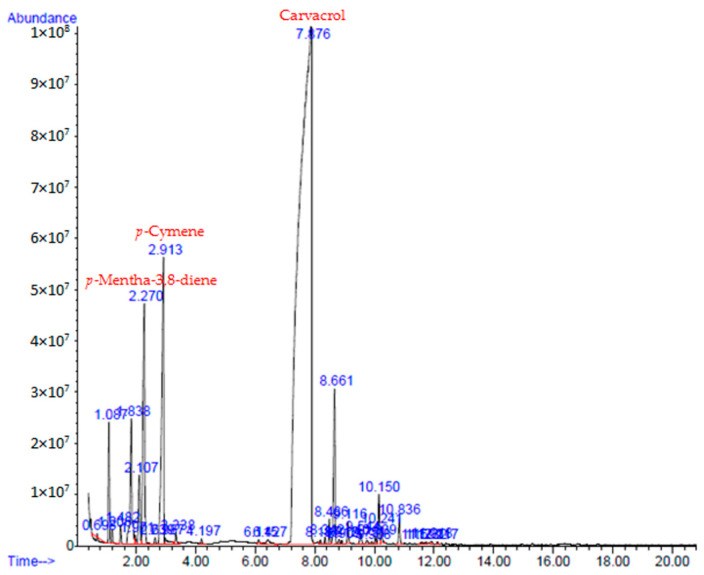
*Origanum compactum* essential oil chromatogram.

Chromatographic analysis of the essential oil of *O. compactum* (OCEO) (Figure 1) identified 32 compounds representing 99.95% of its total chemical composition (Table 1). The main compound was carvacrol (75.70%), followed by mentha-3,8-diene (6.74%), cymene (5.05%), caryophyllene <(E)-> (2.74%), and myrcene (2.71%). The identified chemical compounds of OCEO were separated into oxygenated monoterpenes (76.62%), hydrogenated monoterpenes (17.99%), hydrogenated sesquiterpenes (4.73%), and oxygenated sesquiterpenes (0.61%).

**Figure 2:**
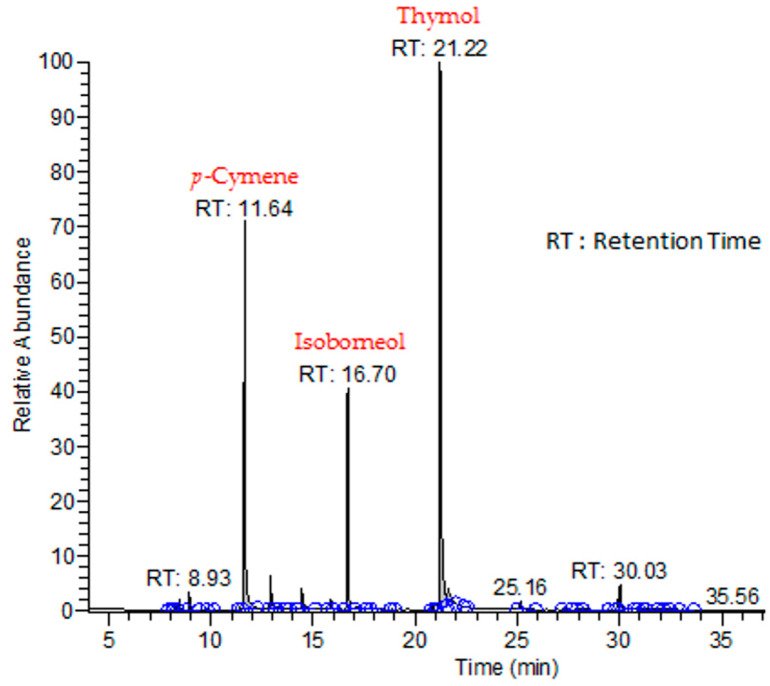
*Thymus zygis* essential oil chromatogram.

Regarding *T. zygis* essential oil (TZEO), the chromatographic analysis (Figure 2) picked out 32 compounds that represent 98.51% of total compounds of the essential oil (Table 1). The three most important compounds accounted for a total of 65.86%, namely thymol (40.67%), *p*-cymene (26.07%), and isoborneol (13.62%). The identified chemical compounds of TZEO were classified into oxygenated monoterpenes (62.52%), hydrogenated monoterpenes (31.31%), oxygenated sesquiterpenes (3.69%), and hydrogenated sesquiterpenes (0.99%). These chemical compositions were putatively determined.

Several studies have reported similar chemical composition for the essential oils of *O. compactum* and have spotlighted that the major compounds were carvacrol, thymol, *p*-mecyne, and γ-terpenine [28,29,30,31,32]. A study that compared the composition of the essential oils extracted from *O. compactum* collected from different regions of Morocco emphasized the presence of the following compounds: α-terpineol (0–25.8%), carvacrol methyl ether (0–36.2%), γ-terpinene (0–35.2%), *p*-cymene (0.2–58.6%), in addition to the two main compounds, thymol (0–80.7%) and carvacrol (0–96.3%) [21]. It was also noted that carvacrol was the predominant component in all the samples collected in the Middle Atlas region. The chemical composition of *O. compactum* essential oils with high percentages of the main compounds cited in the literature corresponds to thymol rich or carvacrol rich chemotypes. The latter refers to plants where the carvacrol content was over 50%, and it has been already indicated for samples of *O. compactum* collected from different regions of Morocco. In particular, Laghmouchi et al. [13] reported that the samples originating from Tetouan, Larache, Ouazzane, and Beni Arous had a high content of carvacrol, which represented 63.95%, 54.84%, 54.21%, and 52.03% of total essential oils, respectively. For the sample growing in Chaouen, carvacrol represented 59% [33], while a similar content of carvacrol (58.1%) was also identified in a sample collected in Rabat [29]. *Origanum compactum* essential oils with content of carvacrol have been also found in samples having their origin from Benslimane, Ouazzane, Oulmes, Taounate, and Moulay Driss Zerhoun, with carvacrol proportions ranging from 90.2% to 96.7% of total essential oils [21]. Very few studies have reported an oregano chemotype characterized by such a high amount of carvacrol. In fact, carvacrol (93.02%) was discovered by Koc et al. [34] to be the main volatile compound in the Turkish *O. bilgeri*, while the same compound was also found in significant amounts, 93.8–95%, in the Greek *O. vulgare* subsp. *hirtum* [35].

In the literature, studies carried out with samples of *T. zygis* collected from different regions of Morocco showed that thymol and carvacrol are the foremost compounds of the essential oil in the respective chemotypes [36,37]. The chemical composition (thymol/*p*-cymene/isoborneol) of *T. zygis* collected for this study is quite similar to the ones collected in another location of Khenifra, such as a sample from Ait Nouh (thymol 38.04%) or the one from Ait Yahia (thymol 32%) [26]. With respect to *T. zygis* studied by Tantaoui et al. [38], its essential oil had a different chemical composition where the main compound was *p*-cymene (50.6%), a precursor of carvacrol, followed by carvacrol (8.1%) and thymol (5%). A small amount of γ-terpinene (2.38%) was also identified, which is known to be the precursor of *p*-cymene. In fact, the harvesting stage could be an explanation for the difference in chemical composition, since the bioconversion of γ-terpinene in *p*-cymene was at its final stage while the bioconversion of *p*-cymene in carvacrol was still in progress, considering that the latter was present at only 2.47%. This is in accord with the previous studies already mentioned, namely that the bioconversion of *p*-cymene in carvacrol occurs between the full flower and the first fruit maturation [39,40].

Considering that the composition of essential oils may be influenced by ecological and genetic factors, the amounts of the main components can vary according to the growing conditions (temperature, humidity, day length, soil type, altitude), as well as according to the nutrients and water availability. That variation also depends on the collection date and the phenological stage of plants at harvesting stage [28,41].

### 2.3. Antioxidant Capacity by DPPH

**Figure 3:**
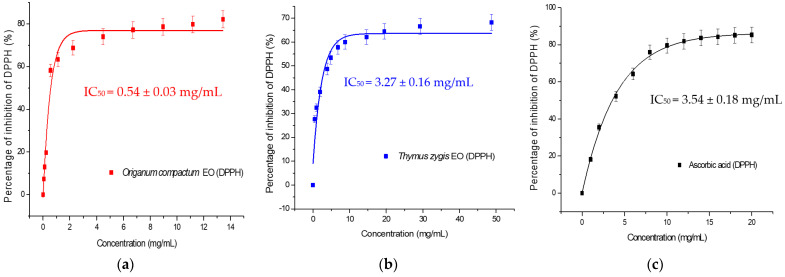
Antioxidant capacity of the essential oils by DPPH (1,1-diphenyl-2-picrylhydrazyl). (**a**) *Origanum compactum* EO; (**b**) *Thymus zygis* EO; (**c**) Ascorbic acid. IC_50_ = Half maximal inhibitory concentration.

Figure 3 presents the results of the antioxidant activity carried out on OCEO and TZEO essential oils, in comparison with ascorbic acid. The recorded antioxidant activity expressed as the concentration of OCEO and TZEO to inhibit 50% of the free radicals of DPPH (IC50) was 0.54 ± 0.03 mg/mL and 3.27 ± 0.16 mg/mL, respectively (Table 2). The reducing power of the free radical DPPH was stronger for OCEO compared to TZEO and ascorbic used as a positive control (IC50 = 3.27 ± 0.16 and 3.54 ± 0.18, respectively).

Regarding OCEO, the results obtained are interesting compared to the ones noted in other studies. In fact, Bouhdid et al. [42] suggested lower antioxidant power (IC50) for samples collected from Boulemane (0.27 ± 0.01 mg/mL) and Taounate (0.37 ± 0.03 mg/mL). Sbayou et al. [43] reported an even higher antioxidant potency with IC50 of 0.021 ± 0.004 mg/mL, which was also higher than the positive control used.

*Thymus zygis* essential oil antioxidant power that we found is similar to the one obtained in previous studies. In fact, a strong antioxidant power of *T. zygis*’s essential oil was recorded with an IC50 of 0.409 ± 0.009 mg/mL for the essential oil of *T. zygis* collected from Ifrane [16] and 0.4 mg/mL for samples of *T. zygis* originated from Portugal [44]. Moreover, Amarti et al. [45] reported a higher DPPH reduction of 0.076 mg/mL for the essential oil of *T. zygis* collected in the region of Middle Atlas, Morocco.

### 2.4. Antioxidant Capacity by FRAP

**Figure 4:**
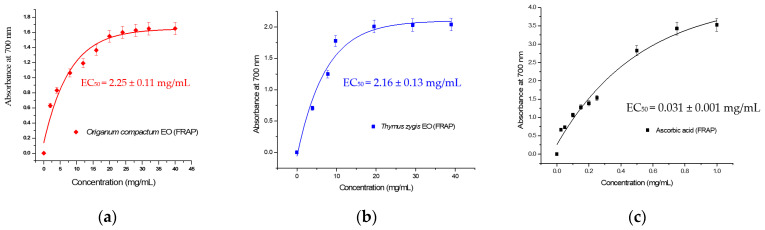
Antioxidant capacity of the essential oils of *Origanum compactum* and *Thymus zygis* assayed by FRAP (Ferric Reducing Antioxidant Power). (**a**) *Origanum compactum* EO; (**b**) *Thymus zygis* EO; (**c**) Ascorbic acid. EC5_0_ = Half maximal effective concentration.

The antioxidant power of the essential oils extracted from *O. compactum* and *T. zygis* was also tested for the ability of those oils to reduce ferrous iron to ferric iron. The results showed that those essential oils possess a significant reduction power but a less important one compared to the one from the ascorbic acid (Figure 4). Actually, the EC50 value for OCEO was around 2.25 ± 0.11 mg/mL compared to 0.031 ± 0.001 mg/mL found for ascorbic acid (Table 2). Al Mijjali et al. [46] noticed a higher antioxidant power with an EC50 of 0.19 ± 0.03 mg/mL and 0.25 ± 0.04 mg/mL for essential oils extracted from *O. compactum* collected from two different regions in Morocco (Boulemane and Taounate). As for TZEO, the antioxidant power was 2.16 ± 0.13 mg/mL, which was feeble compared to the ascorbic acid’s antioxidant power. This low antioxidant activity of TZEO was also observed by Bouymajane et al. [16] for the essential oils of *T. zygis* sampled from Ifrane.

The essential oils of *O. compactum* and *T. zygis* revealed a significant antioxidant activity based on both methods. Their activities were comparable for the FRAP method, whereas the antioxidant activity of OCEO assessed by DPPH method was more powerful than TZEO. This difference could be due to the fact that the studied methods are based on two different chemical reactions. The DPPH method is based on the reaction of hydrogen atom transfer, where the mechanism of action is to remove a hydrogen atom from a donor phenol to produce DPPH-H and a phenoxy radical. On the other hand, the FRAP method is based on the reaction of single electron transfer, and the capacity of the extract to transfer an electron and reduce ferric iron was evaluated.

The antioxidant power of the tested essential oils was closely related to their chemical compositions, especially carvacrol and thymol. According to the literature, several studies have demonstrated that carvacrol and thymol are the main bioactive elements of the essential oils extracted from Lamiaceae species and contribute significantly to their antioxidant power [47,48].

Thymol and carvacrol are isomer monoterpenoid compounds with a single phenolic ring structure with three functional group substituents (hydroxyl group, methyl group and isopropyl group). Their chemical names are 2-isopropyl-5-methylphenol and 5-isopropyl-2-methylphenol, respectively. These compounds are known to be the most prevalent and powerful free radical sensors. They present a system of delocalized electrons due to their hydroxyl, methyl, and isopropyl groups, and they serve as donors for hydrogen or electron allowing the transformation of the radical DPPH• to its reduced form DPPH-H and reducing Fe^3+^ [49]. Therefore, the strong antioxidant activity of the OCEO measured by DPPH method could be attributed to its high content of carvacrol (75.70%). In line with our outcomes, further studies have reported that carvacrol has a better antioxidant activity than thymol independently of the method used to evaluate including DPPH [47,50,51].

### 2.5. Antibacterial Activity

#### 2.5.1. Antibiograms

In relation to the antibiogram illustrated in Table 3, the uppermost antibiotic resistance was observed for the bacterial strains *Pseudomonas* and *Enterobacter* sp. EC3 regarding four out of nine antibiotics tested by exhibiting non-zero inhibition diameters. In contrast, the bacterial strains *Klebsiella pneumoniae* and *Staphylococcus aureus* A1 showed no resistance to any of the nine tested antibiotics. Five bacterial strains were resistant to the least effective antibiotics Ticarcillin (TIM 85) and Trimethoprim (SXT 25), whereas all the bacteria were effectively inhibited by Cefalexin (CN 15) with inhibition zone diameter ranges of 8–20 mm.

Imipenem (IPM 10) and Ciprofloxacin (CIP 5) were the two powerful antibiotics giving the largest inhibition diameters. Imipenem (IPM 10) revealed higher inhibition diameters against *Escherichia coli* strains EC1 and EC3, *Klebsiella pneumoniae*, *Staphylococcus aureus* strains A1, A2, and E. On the other hand, Ciprofloxacin (CIP 5) had the utmost inhibition effect against *Escherichia coli* EC2, *Proteus mirabilis*, and *Pseudomonas* strains.

#### 2.5.2. Disk Diffusion Tests

The antibacterial activity of TZEO and OCEO was evaluated against nine bacterial strains, namely EC1, EC2, EC3, Pseudo, Kleb, Proteus, Staph A1, Staph A2, and Staph E, using the method of diffusion on disk. The antimicrobial effect of two essential oils is greater compared to the most potent antibiotic, especially IPM 10 or CIP 5, except for *Pseudomonas* which was found unaffected toward TZEO. Moreover, the OCEO effect was less pronounced compared to the antibiotic CIP 5 against Pseudo and Proteus (Table 4).

The strongest bacterial activities were expressed by the essential oils of OCEO and TZEO against the bacterial strains *Klebsiella pneumoniae*, *Staphylococcus aureus* A1, and *Staphylococcus aureus* A2 with inhibition zone diameters ranging from 40 to 50 mm. The inhibition zone diameters were greater compared to the ones manifested by the most powerful antibiotics tested, according to the antibiogram presented in Table 3.

The antibacterial power of *O. compactum* essential oil has been already examined and demonstrated in prior studies. In fact, the essential oils of fourteen samples of *O. compactum* collected from various regions of the north of Morocco revealed a significant antibacterial activity against four tested pathogens, *Escherichia coli*, *Bacillus subtilis*, *Listeria innocua,* and *Staphylococcus aureus*, with a diameter of the inhibition zones that ranged from 10.33 to 49.00 mm [13]. This remarkable effectiveness against all the tested strains could be attributed to the high content of carvacrol in the *O. compactum* essential oil. Similarly, the essential oil of *O. compactum* collected from the Rabat area was active toward the strains of *Salmonella enteritidis* and *Salmonella gallinarum* [52]. Also, *O. compactum* essential oils sampled from Boulemane and Taounate were evaluated using the disc diffusion test, and they were very active against *E. coli*, *B. subtilis*, *S. aureus*, and *L innocua* strains. Moreover, the samples collected from Boulemane region showed the uppermost activity due to their highest composition of carvacrol, 45.80% [46].

The antibacterial activity of *T. zygis* essential oil has been studied, particularly its effect against various strains of *L. monocytogenes.* The tests were carried out through the disc diffusion method revealing that the inhibition zone diameters varied between 13.4 ± 0.2 mm and 41.4 ± 0.1 mm [16]. The antibacterial efficacy of *T. zygis* essential oil was also demonstrated against four bacterial strains showing strong inhibition activity against *Bacillus subtilis* and *Micrococcus luteus*, whereas the activity was less significant toward *Escherichia coli* and *Staphylococcus aureus* [45]. Another study investigated the antibacterial activity of essential oils extracted from *T. zygis* and *T. willdenowii* [15]. It was noticed that *T. zygis* essential oil possessed the strongest activity against all the tested pathogens with an inhibition diameter ranging from 6 mm to 84 mm [15]. Ballester-Costa et al. [53] studied the effect of the essential oils of four species of thyme including *T. zygis* against ten bacteria strains and using three different culture media. The results established that *T. zygis* essential oil was the most active in all culture media and was powerful especially against *E. gergoviae* and *L. innocua*.

The essential oils composition is a combination of different components that impact their antibacterial activity. In this respect, the strong antibacterial activities of *O. compactum* and *T. zygis* essential oils recorded in the present study could be attributed to their main components, carvacrol and thymol, respectively. These findings are in concurrence with several studies pointing out the powerful antimicrobial effect of the essential oils rich in carvacrol and/or thymol [13,50,54,55]. In fact, the antibacterial properties of carvacrol and thymol are due to their capacity to disrupt the bacterial membrane, by permeating it and depolarizing it, thus demonstrating antibacterial activity at intracellular sites [56,57,58].

Carvacrol and thymol are phenols with powerful antibiotic effects and are extensively researched for their ability to inhibit a variety of bacteria. Treatment with both compounds inhibits the development of harmful organisms such as *Esherichia coli*, *Listeria monocytogenes*, and *Salmonella enterica* subsp. *enterica* serovar Typhimurium, in addition to *Shigella sonnei* and *Staphylococcus aureus* [8]. Thymol and carvacrol with their hydroxyl, methyl, and isopropyl groups present a system of delocalized electrons that plays a crucial role on the antibacterial activities. These double bonds of electrons lead carvacrol and thymol to function proton exchanger, which reduces the gradient across the cytoplasmic membrane and causes the collapse of the proton motive force and the depletion of the ATP pool, which ultimately leads to cell death [8].

However, the antibacterial power of an essential oil should not be attributed only to the main components but also to its chemical composition that may interact with each other. These interactions can be additive, synergistic, or antagonistic. It should be noted that less prevalent components may significantly contribute to the antibacterial activity of the whole essential oil. In fact, research studies have shown that the actual essential oil has a greater biological power than the combination of the isolated major compounds [59,60]. According to the same studies, even minor compounds are essential to the biological activity revealed by the essential oil [59,60], while the combination of thymol and carvacrol in the same essential oil produces an additive effect [13,61].

*P*-cymene, the second foremost compound of *T. zygis* and *O. compactum* essential oils, is the precursor of carvacrol, and it has been mentioned to have a weak antibacterial power [40,55]. Nevertheless, it also plays a significant role in the overall antibacterial activity of the essential oil. It acts in synergy with carvacrol and thymol by facilitating their intracellular penetration, and thus it increases their antibacterial power [57]. According to Ultee et al. [57], the cytoplasmic membrane swells easily when *p*-cymene, a hydrophobic molecule, is present in the essential oil composition compared to the essential oil having only carvacrol. The importance of *p*-cymene lies in its ability to integrate the bacterial lipid layer and assist the transport of carvacrol across the cytoplasmic membrane, which maximizes the essential oil’s efficiency. Borneol has also been identified in TZEO, and its role in the antibacterial activity is not negligible. In fact, due to its great solubility in water, borneol has a strong ability to penetrate bacterial cell membranes, making it a substance with a high antibacterial potency [62,63].

The nature of bacteria influences the effect of the essential oils as well. Because of the design of their outer membrane, Gram-negative bacteria are typically more resistant than Gram-positive bacteria. The outer membrane of Gram-negative bacteria is richer in proteins and lipopolysaccharides than Gram-positive bacteria, which make them more hydrophilic and less adhesive to hydrophobic terpenes. Some membranes can be broken through by some low molecular weight phenolic compounds, such as carvacrol and thymol, which are easily attached to lipopolysaccharides and membrane proteins through their functional groupings [13].

## 3. Materials and Methods

### 3.1. Origin of Plant Samples

*Origanum compactum* and *Thymus zygis* samples were collected in Khenifra, a region located in the mountains of Middle Atlas in Morocco. The *Origanum compactum* was cultivated while *T. zygis* was collected from the peripheries of Khenifra. The collected samples were dried for 10 days in the shade at room temperature. The Scientific Institute of Rabat, Morocco, carried out the plant’s botanical identification and voucher specimens were deposed in the Herbarium under the references RAB114608 (for *Thymus zygis*) and RAB114609 (for *Origanum compactum*).

### 3.2. Extraction and Analysis of Essential Oils

Hydrodistillation with Clevenger apparatus was used to extract the essential oils. For each plant, dried plant material (100 g) and water (1 L) were boiled for at least 3 h. Essential oils obtained were kept at 4 °C in complete darkness. The yield (%*y*) of essential oil extracted was calculated from moisture content (*MC*), the volume of essential oil (*V*), and the weight of the plant (*m*0) used to extract.

(1) $$\%y=\frac{V}{m0-(m0\times \%MC)}\times 10^{4}$$

The analysis of the chemical composition of the essential oil was performed by gas chromatography (GC) coupled with a mass spectrometer (MS). It was implemented on a Thermo Scientific™ TRACE™ 1310 GC equipped with DB-5 capillary column (30 m × 0.25 mm, film thickness 0.25 μm) (5% phenyl-methyl-siloxane), coupled to Thermo Finnigan POLARISQ Ion Trap Mass Spectrometer (Thermo Fisher Scientific, Waltham, MA, USA). 

The chromatographic conditions were as follows: Injector and detector temperatures at 220 and 300 °C, respectively; carrier gas is nitrogen with a flow rate of 1 mL/min; temperature programming ranges from 50 to 200 °C for 5 min, with a gradient of 4 °C/min; injected volume is 1 μL.

The identification of the chemical composition of the essential oils was based on the comparison of the Linear Retention Index along with those references known in the literature [64,65]. It was supplemented by a comparison of indices and mass spectra, obtained by gas chromatography coupled with mass spectrometry (GC/MS), with different references [65]. The Linear Retention Index compares the retention time of any product with that of a linear alkane of the same carbon number. They were defined by injecting a mixture of alkanes (standard C7–C40) under the same operating conditions. The Linear Retention Index (called also Kovats indices KI) was calculated using this equation:

(2) $$KI=[\frac{{TR}_{x}-{TR}_{n}}{{TR}_{n+1}-{TR}_{n}}+n]\times 100$$

where ${TR}_{x}$ is the retention time of the solute *x*; and ${TR}_{n}$ and ${TR}_{n+1}$ are the retention times of linear alkanes. Quantification of compounds was expressed as relative percentages based on the peak areas of chromatographs and the use of internal standards of known concentration.

### 3.3. Antioxidant Activity

Two techniques, DPPH and FRAP, were employed to estimate the antioxidant capacity, based on protocols described in the literature [6]. The first approach assesses the ability of the 1,1-diphényl-di-picrylhydrazyl (DPPH) radical to trap hydrogen atoms, while the second method, FRAP, is founded on the single electron transfer reaction and assesses the antioxidant capacity of ferric iron (Fe^3+^) reduction to ferrous iron (Fe^2+^).

#### 3.3.1. Test of Antioxidant Capacity by DPPH

An ethanolic solution of DPPH was prepared using 2.4 mg of DPPH in 100 mL of ethanol. Then, a stock solution with concentrations of 224 mg/mL for OCEO and 195 for TZEO was prepared by adding 800 µL of ethanol to 200 µL of each oil OCEO and TZEO. A series of dilutions were prepared from this mother solution. The tests were carried out by mixing different concentrations EOS solution (200 µL) with 2.8 mL of the DPPH solution. After 30 min in complete darkness, the absorbance was measured at 517 nm. As a positive control, the effect that ascorbic acid had on the free radical DPPH was also examined in the same conditions.

The antioxidant power was estimated by the parameter IC50, which represents the amount of antioxidant needed to reduce a compound’s initial concentration by 50%. The lower its value, the more significant the compound’s antioxidant capacity.

#### 3.3.2. Test of Antioxidant Capacity by FRAP

The test of antioxidant capacity by FRAP is based on a reduction in ferric ions (Fe^3+^), given by potassium ferricyanide (K_3_Fe(CN)_6_), to ferrous ions (Fe^2+^) by the antioxidants in EOs. Several dilutions of the essential oils were prepared with different concentrations ranging between 0 and 50 mg/mL. Ascorbic acid was also tested under the same conditions as a positive control. The antioxidant power was estimated through the effective concentration, (EC50), which is associated with an absorbance of 0.5, inversely related to the compound’s antioxidant capacity [6].

### 3.4. Antibacterial Activities

#### 3.4.1. Setting up Strains of Bacteria

To evaluate their antibacterial activity, nine bacterial strains belonging to six species were tested: *Escherichia coli* (EC1, EC2, EC3), *Pseudomonas aeruginosa* (Pseudo), *Klebsiella pneumonia* (Kleb), *Proteus mirabilis* (Proteus), *Staphylococcus aureus* (Staph A1, Staph A2), and *Staphylococcus epidermidis* (Staph E). Bacterial strains were refreshed in Petri dishes with the Mueller–Hinton broth. Then, a sterile swab of a bacterial colony was used to create the inoculum. It was decanted into a tube with sterile physiological water (2.5 mL) and then it was vigorously shaken. To obtain the same concentration of bacteria for each bacterium, four boxes were seeded with the same inoculum. Once the boxes were dried, one was used to create the antibiogram, and the other three were used to calculate the essential oils’ antibacterial activity. Then, the inhibitory zones’ average from three replicates (n = 3) was determined.

#### 3.4.2. Antibiograms

The antibiotic effect profiles of the strains were carried out according to the guidelines of EUCAST (the European Committee on Antimicrobial Susceptibility Testing) [66] and the French Microbiological society [67]. A disk distributor was used to perform the antibiogram. Nine disks containing various antibiotics (ATB) (Amoxicillin, Cefalexin, Ceftriaxone, Ciprofloxacin, Doxycycline, Imipinem, Ofloxacin, Ticracillin, Trimethoprim) were tested. The dishes were incubated for 24 h at 37 °C in a steam chamber. The diameter of inhibition zone was then measured.

#### 3.4.3. Disk Diffusion Tests

Four (4) μL of essential oil was added to each dish seeded with the identical inoculum. The dish was incubated at 37 °C for 24 h in the steam chamber. Each diameter of inhibition zone was measured, and the results were compared with the corresponding diameter measured in the antibiogram.

### 3.5. Statistical Analysis

The statistical analyses were performed using SPSS 22 (IBM, Armonk, NY, USA) and Origin 9.2 (OriginLab Corporation, Northampton, MA, USA) software programs. The means ± standard errors were used to express the obtained results. A one-way analysis of variance (ANOVA) was used in the statistical analysis of the antioxidant capacity. The differences were deemed significant at *p* ≤ 0.05, and each experiment was carried out three times.

## 4. Conclusions and Perspectives

The essential oils extracted from two plants belonging to Lamiaceae family, *O. compactum* and *T. zygis*, were analyzed and tested to evaluate their antioxidant and antibacterial activities. Carvacrol (75.70%) and thymol (40.67%) were found to be the main compounds of the chemical composition of *O. compactum* and *T. zygis* essential oils, respectively.

The antioxidant capacity of the two essential oils was tested using DPPH and FRAP methods. *Origanum compactum* essential oil was found to be more potent than ascorbic acid used as a positive control. Regarding the antibacterial activity, the essential oils were more effective than most of the tested antibiotics, mainly due to their high content in carvacrol and thymol. Overall, this study exhibits initial results about the antioxidant and antibacterial potentials of *O. compactum* and *T. zygis* collected from the Middle Atlas of Morocco. Also, we identified the main compounds for each essential oil involved in the antioxidant and antibacterial activities. This might suggest the versatile applications of *O. compactum* and *T. zygis* essential oils in the pharmaceutical, cosmetic, and food industries to substitute synthetic antioxidants and antibiotics. However, more in-depth investigations on bactericidal and inhibitory concentration, toxicity, and safety conditions are required for the use these two essential oils, while further research is needed to explore more bioactivities. This study hopes to shed light on these endemic species widely consumed in this Moroccan Middle Atlas region for better valorization, ensuring the sustainability of exploitation for the local population.

## Figures and Tables

**Table 1:** Chemical composition of *Origanum compactum* and *Thymus zygis* essential oils.

Retention Time	Linear Retention Index	Compound	% OCEO	Retention Time	Linear Retention Index	Compound	% TZEO
1.085	930	α-Thujene	1.29	8.28	930	α-Thujene	0.16
1.203	954	Camphene	0.12	8.45	939	α-Pinene	0.26
1.482	975	Sabinene	0.24	8.93	954	Camphene	1.18
1.836	990	Myrcene	2.71	9.91	1011	δ-3-Carene	0.12
2.105	1002	δ-2-Carene	1.27	11.40	1017	α-Terpinene	0.26
1.976	1011	δ-3-Carene	0.15	11.64	1024	*p*-Cymene	26.07
2.266	1024	*p*-Cymene	5.05	12.91	1059	γ-Terpinene	2.90
2.641	1059	γ-Terpinene	0.10	13.22	1070	Cis-Sabinene hydrate	0.43
2.985	1070	Cis-Sabinene hydrate	0.25	14.04	1090	Linalool (dihydro)	0.09
2.910	1072	*p*-Mentha-3,8-diene	6.74	14.44	1096	Linalool	2.42
3.339	1088	Terpinolene	0.22	15.86	1126	α-Campholenal	0.73
4.198	1132	allo-Ocimene	0.10	16.04	1127	Chrysanthenone	0.13
6.431	1188	α-Terpineol	0.23	18.87	1129	γ-Terpineol	0.11
6.120	1235	Thymol, methyl ether	0.07	16.70	1160	Isoborneol	13.62
7.880	1299	Carvacrol	75.70	17.15	1171	Isopulegol	0.54
8.342	1372	Carvacrol acetate	0.09	17.71	1182	*p*-Cymen-8-ol	0.11
8.481	1385	6-Allyl-2-cresol	0.28	17.89	1184	Thuj-3-en-10-ol	0.23
8.192	1388	β-Bourbonene	0.07	20.87	1285	Bornyl acetate	0.34
8.664	1419	Caryophyllene(E)	2.74	21.22	1290	Thymol	40.67
8.814	1432	β-Copaene	0.08	21.61	1299	Carvacrol	3.01
8.911	1441	Aromadendrene	0.04	22.46	1352	Thymol acetate	0.09
9.115	1454	α-Humulene	0.33	25.16	1408	Caryophyllene (Z)	0.73
9.748	1496	Viridiflorene	0.10	28.04	1515	10-epi-Italicene ether	0.15
9.544	1513	γ-Cadinene	0.23	28.35	1528	Cis-Calamenene	0.26
10.038	1513	γ-Cadinene	0.11	29.91	1578	Spathulenol	0.71
9.909	1500	α-Muurolene	0.04	30.03	1583	Caryophyllene oxide	2.04
10.145	1505	β-Bisabolene	0.73	30.99	1619	1,10-di-epi-Cubenol	0.10
10.242	1523	δ-Cadinene	0.26	32.20	1646	Cubenol	0.07
10.832	1583	Caryophyllene oxide	0.43	31.64	1649	Cis-Guaria-3,9-dien-11-ol	0.23
11.595	1640	Caryophylla-4(12), 8(13)-dien-5α-ol	0.05	32.10	1653	Himachalol	0.12
11.734	1640	epi-α-Cadinol	0.06	33.32	1667	14-hydroxy-9-epi Caryophyllene Z	0.16
11.917	1689	Shyobunol	0.07	32.57	1669	14-hydroxy-9-epi Caryophyllene E	0.11
Total (%)			99.95	Total (%)			98.51

OCEO: *Origanum compactum* essential oil; TZEO: *Thymus zygis* essential oil.

**Table 2:** Values of the antioxidant activity (IC_50_ and EC_50_) for OCEO, TZEO, and ascorbic acid.

	OCEO	TZEO	Ascorbic Acid
IC_50_ (mg/mL)	0.54 ± 0.03	3.27 ± 0.16	3.54 ± 0.18
EC_50_ (mg/mL)	2.25 ± 0.11	2.16 ± 0.13	0.031 ± 0.001

OCEO: *Origanum compactum* essential oil; TZEO: *Thymus zygis* essential oil. IC_50_ = Half maximal inhibitory concentration. EC5_0_ = Half maximal effective concentration.

**Table 3:** Antibiotic inhibition zone results expressed in mm.

	Bacteria	*Escherichia coli* 1	*Escherichia coli* 2	*Escherichia coli* 3	*Pseudomonas* sp.	*Klebsiella pneumoniae*	*Proteus mirabilis*	*Staphylococcus aureus* A1	*Staphylococcus aureus* A2	*Staphylococcus epidermidis*
ATB										
AMC 30		8 ± 0.4	0.5 ± 0.02	14 ± 0.7	0 ± 0.01	14 ± 0.7	0 ± 0.01	34 ± 1.6	22 ± 1.1	0 ± 0.01
CN 15		12 ± 0.6	12 ± 0.6	10 ± 0.5	8 ± 0.4	14 ± 0.7	14 ± 0.7	20 ± 1	14 ± 0.5	8 ± 0.4
CRO 30		8 ± 0.4	22 ± 1.1	0 ± 0.01	0 ± 0.01	28 ± 1.4	22 ± 1.1	12 ± 0.6	12 ± 0.6	1 ± 0.05
CIP 5		0 ± 0.01	**28 ± 1.3**	0 ± 0.01	**30 ± 1.5**	26 ± 1.3	**34 ± 1.6**	28 ± 1.4	20 ± 1	12 ± 0.6
DO 30		10 ± 0.5	7 ± 0.3	10 ± 0.4	0 ± 0.01	20 ± 1	17 ± 0.85	36 ± 1.8	18 ± 0.9	2 ± 0.1
IPM 10		**26 ± 1.2**	26 ± 1.3	**22 ± 1.1**	24 ± 1.2	**30 ± 1.5**	0 ± 0.01	**38 ± 1.9**	**42 ± 1.9**	**15 ± 0.7**
OFX 5		0 ± 0.01	26 ± 1.3	0 ± 0.02	20 ± 1	24 ± 1.2	26 ± 1.3	30 ± 1.5	24 ± 1.2	11 ± 0.55
TIM 85		0 ± 0.02	0 ± 0.01	0 ± 0.02	0 ± 0.01	16 ± 0.8	14 ± 0.7	20 ± 1	12 ± 0.6	0 ± 0.01
SXT 25		0 ± 0.02	0 ± 0.01	0 ± 0.01	0 ± 0.01	22 ± 1.1	19 ± 0.95	26 ± 1.3	0 ± 0.03	8 ± 0.4

AMC: Amoxicillin, CN: Cefalexin, CRO: Ceftriaxone, CIP: Ciprofloxacin, DO: Doxycycline, IPM: Imipenem, OFX: Ofloxacin, TIM: Ticarcillin, SXT: Trimethoprim. Bold cells indicate the highest diameter of inhibition. ATB: Antibiotics.

**Table 4:** Inhibition zones measurements of OCEO and TZEO in mm.

	Bacteria	*Escherichia coli* 1	*Escherichia coli* 2	*Escherichia coli* 3	*Pseudomonas* sp.	*Klebsiella pneumoniae*	*Proteus mirabilis*	*Staphylococcus aureus* A1	*Staphylococcus aureus* A2	*Staphylococcus* *epidermidis*
EOs and ATB+										
OCEO		36 ± 1.8	30 ± 1.5	32 ± 1.6	14 ± 0.7	40 ± 2	25 ± 1.2	52 ± 2.6	46 ± 2.3	28 ± 1.2
TZEO		36 ± 1.8	34 ± 1.7	36 ± 1.6	0 ± 0.02	40 ± 2	36 ± 1.6	50 ± 2.5	46 ± 2.3	20 ± 1
ATB^+^		26 ± 1.2 IPM 10	28 ± 1.3 CIP 5	22 ± 1.1 IPM 10	30 ± 1.5 CIP 5	30 ± 1.5 IPM 10	34 ± 1.6 CIP 5	38 ± 1.9 IPM 10	42 ± 1.9 IPM 10	15 ± 0.7 IPM 10

OCEO: *Origanum compactum* essential oil; TZEO: *Thymus zygis* essential oil. EOs: Essential oils; ATB: Antibiotics. IPM: Imipenem, CIP: Ciprofloxacin.

## Data Availability

Data are contained within the article.
